# Use of atypical antipsychotics for post-hospitalization delirium and associated clinical outcomes in older adults

**DOI:** 10.1093/geroni/igaf122.1652

**Published:** 2025-12-31

**Authors:** Chun-Ting Yang, James Wilkins, Kevin Pritchard, Xiaojuan Liu, Dae Hyun Kim, Joshua Lin

**Affiliations:** Brigham and Women’s Hospital, Boston, Massachusetts, United States; McLean Hospital, Belmont, Massachusetts, United States; Brigham and Women’s Hospital, Boston, Massachusetts, United States; Brigham and Women’s Hospital, Boston, Massachusetts, United States; Hebrew SeniorLife, Boston, Massachusetts, United States; Brigham and Women’s Hospital, Boston, Massachusetts, United States

## Abstract

Atypical antipsychotic medications (APMs) are commonly prescribed to manage delirium symptoms among hospitalized older adults, but their comparative safety beyond hospital discharge remains unknown. This retrospective cohort study utilized Medicare 2013-2018 and Optum Clinformatics® 2013-2024 to examine the risks of clinical outcomes between quetiapine, risperidone, and olanzapine use among older adults following hospitalization. We included patients aged≥65 years without psychiatric disorders who newly filled quetiapine, risperidone, or olanzapine within 30 days of hospital discharge. The index date was the first dispensing date of the APM. Propensity score overlap weighting was employed to balance baseline covariates. Outcomes included all-cause mortality, all-cause rehospitalization, and specific rehospitalization causes. The on-treatment effects were measured using Poisson regression model. Analyses were conducted in the two databases separately and the effect estimates were pooled by fixed-effect meta-analysis. A total of 32,209, 9,732, and 6,363 quetiapine, risperidone, and olanzapine users were identified. The mortality rate (per 1000 person-years) was 478.1 for quetiapine (reference), 544.3 for risperidone (rate ratio [95% CI]: 1.11 [0.96-1.29]), and 726.2 for olanzapine (1.49 [1.30-1.71]). The rehospitalization rate was 1,408.6 for quetiapine (reference), 1,540.6 for risperidone (1.07 [0.97-1.17]), and 1,703.2 for olanzapine (1.16 [1.06-1.29]). Olanzapine also showed a significantly higher risk of death (1.34 [1.17-1.53]) compared to risperidone. Among rehospitalization reasons, olanzapine was associated with an increased risk of delirium compared to quetiapine (1.34 [1.12-1.60]) and risperidone (1.20 [1.01-1.42]). Our findings suggest that olanzapine increased the risks of adverse outcomes compared to risperidone and quetiapine among older adults prescribed with APMs after hospital discharge.

